# Modern Sociology

**Published:** 1899-09-16

**Authors:** 


### Modern Sociology.

### THE ETHICS OF SUICIDE.—III.

By Francis Scougall.

In attempting to diagnose the mental maladies which by some secret and occult power impel men to fling away the unique gift of life, we are certainly, as I have said, dealing with some of the most mysterious instincts of human nature. That the motives for an act which, viewed in its simplest aspect, can only be characterised as one of preposterous folly, spring from conditions which are totally different in the case of the educated classes from those in a lower grade of society does not render the whole subject a less bewildering problem than it really is, but I can only hope to throw light on any possible means of lessening the prevailing tendency to voluntary euthanasia, by seeking to elucidate its causes. I am still at present dealing with the lowest ranks of the people, and one of the most common forms of the impulses which drive them to self-destruction seems very clearly to refute Shakespeare's assertion that "Men have died and worms have eaten them, but not for love"—and yet it may be that this statement of his, in spite of its apparent fallacy, does but make his extraordinary penetration into the intricacies of human action still more strikingly manifest; for although in the great majority of suicides perpetrated by young people disappointed affections appear to be the obvious cause creating the desire to quench all feeling, there is an underlying motive which in most cases is the real determining agency in the act;—that is the desire of revenge; whether it be a man or a woman whose passionate love has been perhaps favourably received at first, and then finally rejected and scorned, the bitterness of pain and humiliation, the burning indignation against the deceiver who has flung them from a joyful height of hope and longing, to grovel in a dreary solitude, turns to the desire in lower natures to take vengeance for their own sufferings by inflicting all the pain and anguish they can by any means create on the author of their own misery. They know of no better means to compass this end than by bringing down on them an intolerable remorse which can never be assuaged, because it has its root in death. They know well that there are few, even of the coarsest type, who could look without strong regret and pain on the cold, lifeless form of one they had last seen in the full vigour and promise of life, animated by a flattering ardent passion for themselves, and to know—probably by written words stating the fact mercilessly—that their own falseness or self-interest has caused their victim to drop from hope and sunshine to the hidden darkness of an untimely grave. It is, therefore, not disappointed love, but the mad desire to be revenged on a scornful maid or man, that sends into oblivion many who are supposed to die from unrequited affection.

In this category of uneducated suicides we see very markedly the singular indifference with which such hasty-tempered persons treat the possible future that might have been theirs on earth, and the unknown conditions that may await them in a future sphere. Not a glance do they cast to these contingencies, not a thought do they give to the fact that their twenty or thirty years of life might have stretched to eighty, full of great possibilities, nor do they for a second think of the many voices echoing round them with pregnant intimations concerning an unseen momentous future, waiting every spirit that passes its probation here. No! the present is their all of existence, according to their own recognition of their position; for the time it is dominated by the galling certainty that they have been duped and despised, and the one who lacerated their heart and their vanity shall suffer pangs of vain regret at the cost of their own destruction.

This entire absence of any inclination to look beyond the existing moments characterises in an extraordinary manner—all those with whom I, at least, have come in contact—among suicides of the lower classes, and it forms one of the grounds on which, I think, some attempt might be made to grapple with the subject, on the part of those who would be qualified for the arduous and difficult effort it could not fail to be. This same determination not to look into the future which prevails almost universally among lower-class suicides, obtains also very largely among those of a higher order of intellect, to whom we must now turn, although in their case the conditions from which it results are in most respects quite dissimilar. We enter now upon a very different phase in the mysterious ethics of suicide, when we turn to regard it as it operates on those who are surrounded with the luxuries and pleasures of life, who have never known what it is to have a want ungratified, for whom discomfort or an absence of ease and well-being can only be caused by actual physical pain, and who, in a word, so far as outward conditions are concerned, would seem to be as little likely to desire an abrupt and overwhelming change as Dives; he faring sumptuously every day could look down calmly upon the beggar that might not unnaturally have desired to escape his cold position at the rich man's gate—barely existing on the scanty insufficient crumbs, and the ministrations of the dogs more merciful than man. Of course, this immunity from want and squalid misery is the very lowest ground which would seem to exempt persons of means and refinement from a tendency to suicide, for there are artistic and intellectual pleasures which might be thought almost overwhelmingly attractive to men of culture apart from grosser enjoyments, which in various shapes are within the reach of all. I admit, of course, that the weird strokes of destiny can render life so appallingly desolate as to make it appear an intolerable burden, but side by side with this most obvious truth there is a deterrent force against the embrace of death, to which the discoveries of science have of late years added an enormous weight: my nearly exhausted space at present permits me to name only one. But there is much to be said on this fruitful subject in the future. Scientific researches into the constitution of human nature have greatly deepened the mysteries of life and death, and, amongst other occult facts which have arisen from these discoveries, it has been found impossible to determine when dissolution actually takes place. According to some it is only when decomposition has set in to an unmistakeable extent that certainty can be obtained. The fear, therefore, of being buried alive is one which may be imagined to appeal to those who contemplate suicide in an infinitely greater degree than has hitherto been the case.

